# Effects of mHealth Interventions on Improving Antenatal Care Visits and Skilled Delivery Care in Low- and Middle-Income Countries: Systematic Review and Meta-analysis

**DOI:** 10.2196/34061

**Published:** 2022-04-22

**Authors:** Md Obaidur Rahman, Noyuri Yamaji, Yasuko Nagamatsu, Erika Ota

**Affiliations:** 1 Department of Global Health Nursing Graduate School of Nursing Science St. Luke's International University Tokyo Japan; 2 Center for Surveillance, Immunization, and Epidemiologic Research National Institute of Infectious Diseases Tokyo Japan; 3 Tokyo Foundation for Policy Research Tokyo Japan

**Keywords:** mobile health, ANC, skilled delivery care, LMICs, systematic review and meta-analysis

## Abstract

**Background:**

The poor coverage of essential maternal services, such as antenatal care (ANC) and skilled delivery care utilization, accounts for higher maternal and infant mortality in low- and middle-income countries (LMICs). Although mobile health (mHealth) interventions could potentially improve the service utilization in resource-limited settings, their effectiveness remains unclear.

**Objective:**

This review aimed to summarize the effect of mHealth interventions on improving the uptake of ANC visits, skilled birth attendance at the time of delivery, and facility delivery among pregnant women in LMICs.

**Methods:**

We conducted a comprehensive search on 9 electronic databases and other resources from inception to October 2020. We included individual randomized controlled trials and cluster randomized controlled trials that assessed the effectiveness of mHealth interventions for improving perinatal health care utilization among healthy pregnant women in LMICs. We performed a random-effects meta-analysis and estimated the pooled effect size by using risk ratios (RRs) with 95% CIs. In addition, 2 reviewers independently assessed the risk of bias of the included studies by using the Cochrane risk of bias tool and the certainty of the evidence by using the Grading of Recommendation, Assessment, Development and Evaluation approach.

**Results:**

A total of 9 studies (10 articles) that randomized 10,348 pregnant women (n=6254, 60.44% in the intervention group; n=4094, 39.56% in the control group) were included in this synthesis. The pooled estimates showed a positive effect of mHealth interventions on improving 4 or more ANC visit utilizations among pregnant women in LMICs, irrespective of the direction of interventions (1-way communications: RR 2.14, 95% CI 1.76-2.60, *I*^2^=36%, 2 studies, moderate certainty; 2-way communications: RR 1.17, 95% CI 1.08-1.27, *I*^2^=59%, 3 studies, low certainty). Only 2-way mHealth interventions were effective in improving the use of skilled birth attendance during delivery (RR 1.23, 95% CI 1.14-1.33, *I*^2^=0%, 2 studies, moderate certainty), but the effects were unclear for 1-way mHealth interventions (RR 1.04, 95% CI 0.97-1.10, *I*^2^=73%, 3 studies, very low certainty) when compared with standard care. For facility delivery, the interventions were effective in settings where fewer pregnant women used facility delivery (RR 1.68, 95% CI 1.30-2.19, *I*^2^=36%, 2 studies, moderate certainty); however, the effects were unclear in settings where most pregnant women already used facility delivery (RR 1.01, 95% CI 0.97-1.04, *I*^2^=0%, 1 study, low certainty).

**Conclusions:**

mHealth interventions may contribute to improving ANC and skilled delivery care utilization among pregnant women in LMICs. However, more studies are required to improve their reproducibility and efficiency or strengthen the evidence of different forms of mHealth interventions because of the considerable heterogeneity observed in the meta-analyses.

**Trial Registration:**

PROSPERO CRD42020210813; https://tinyurl.com/2n7ny9a7

## Introduction

### Background

Despite progress in improving global maternal mortality, it remains unacceptably high, particularly in low- and middle-income countries (LMICs) [1]. Reducing complications during and following pregnancy and childbirth, when most complications occur, can reduce or prevent maternal mortality. Skilled care during and following pregnancy and childbirth could reduce complications and may result in preventing maternal deaths. Studies conducted in Tanzania and Ethiopia have confirmed the ability of antenatal care (ANC) and skilled birth attendance (SBA) during labor and delivery to reduce maternal mortality [2-4].

Globally, most pregnant women have access to ANC with a skilled health professional (eg, physician, nurse, or midwife) at least once, but only 65% receive the World Health Organization–recommended number of at least four ANC visits, and 81% of births occur with the assistance of skilled health personnel [5]. Although there has been a significant improvement in the coverage for SBA and facility delivery in the last decade, millions of births still occur annually without any assistance from a skilled health professional [6]. Several factors prevent pregnant women from receiving the care provided by skilled health personnel during pregnancy and childbirth, such as lack of information, limited preventive health education, limited access to maternal health services owing to poverty or distance factors, poor administration, shortage of health care professionals, and inadequate or poor-quality services [7,8].

In LMICs, only approximately half of pregnant women receive 4 or more ANC visits, and the rate of skilled delivery care including SBA and facility delivery is relatively poor [5]. Moreover, the lowest levels of ANC and skilled delivery coverage are observed in regions where maternal mortality remains excessively high. For instance, the coverage for 4 or more ANC visits was 49% in South Asia and 52% in sub-Saharan Africa [5]. In terms of SBA at the time of delivery, the coverage was 60% in sub-Saharan Africa and 77% in Southern Asia, whereas other World Health Organization regions have achieved universal coverage [5]. The poor coverage of ANC and SBA accounted for the higher maternal mortality in these regions. Hence, a faster pace of progress is required to improve the coverage of ANC and skilled delivery in these high-burden regions.

In the last decade, mobile phone coverage has rapidly increased worldwide. The International Telecommunication Union reported that global mobile phone subscriptions crossed >7 billion in 2015, and mobile phone penetration reached over 90% in LMICs [9]. Thus, mobile phone penetration has the potential to strengthen existing health care service utilization, particularly ANC, SBA, and facility delivery services, in resource-limited settings in a cost-effective manner. Mobile health (mHealth) interventions are becoming more widespread in LMICs because the technology involved is more rapid and accessible than internet access. Labrique et al [10] reported on 12 mHealth applications that could respond to various health issues. Most mHealth interventions are designed to promote behavior change in patients or health personnel by providing health care reminders, health advice, health education, health information and facilitating referral, or access to health facilities or point-of-care remote consultation. Numerous models of mHealth interventions have been used to support pregnant women during and following pregnancy and childbirth in LMICs [11]. Previous studies have reported that mHealth interventions may be capable of and effective in improving essential maternal health care service utilization in LMICs [12-21]. Most of the systematic reviews narratively synthesized the available literature and reported a great potential for mHealth interventions to change maternal health care–seeking behaviors and showed a positive effect on improving ANC, SBA, postnatal care, or childhood immunization [12,14,15,18,22]. However, most evidence comes from observational studies and pilot or small-scale mHealth intervention studies, and researchers have expressed concerns about the study quality [12,14,15,18,22].

Although mHealth interventions have shown a great potential for behavior change more broadly, there are relatively few rigorous evaluations assessing the effectiveness of mHealth interventions on the uptake of ANC, SBA, and facility delivery utilization among pregnant women [19,21]. A recent systematic review reported a significantly higher mean of ANC attendance in mHealth interventions than standard care, but the result remains inconclusive because of the higher statistical heterogeneity among the studies; they also summarized randomized controlled trials (RCTs) and non-RCTs together into the meta-analysis [21]. Another systematic review considered RCTs to rigorously evaluate the effectiveness of mHealth interventions, and a meta-analysis based on a limited number of studies found a positive effect on improving 4 or more ANC visits and SBA outcomes [19]. Owing to higher statistical heterogeneity and the limited number of studies for each outcome, the evidence remains inconclusive [19]. To the best of our knowledge, no systematic review has reported on the effectiveness of mHealth interventions in improving facility delivery outcomes. Therefore, a rigorous evaluation of high-level studies is required to assess the effects of mHealth interventions on ANC and skilled delivery care utilization (SBA and facility delivery) in LMICs. This review further summarizes the findings of high-level studies (such as RCTs) and provides a clear direction to health practitioners, researchers, and policy makers.

### Objectives

The objective of this review is to explore and synthesize the effects of mHealth interventions on improving the uptake of ANC visits, SBA at the time of delivery, and facility delivery among healthy pregnant women in resource-limited settings.

## Methods

### Protocol Registration and Review Guideline

The review protocol was registered in the PROSPERO database (CRD42020210813) [23]. The guideline of PRISMA (Preferred Reporting Items for Systematic Reviews and Meta-Analyses) was followed for reporting this systematic review and meta-analysis [24].

### Search Methods for Study Identification

Using a highly sensitive search strategy, we conducted a comprehensive search from inception to October 2020 to identify RCTs, including cluster RCTs, in the following electronic databases: APA PsycINFO, British Nursing Index, CINAHL Plus, Embase, MEDLINE, POPLINE, PubMed, The Cochrane Library, and Web of Science. We used controlled vocabulary and text words for each database. The search terms were grouped into three major categories of interest: participants (pregnant women), interventions (mHealth interventions), and study designs (RCTs). We did not limit our search to language, date, or publication type to include all published studies. Moreover, we checked the reference lists of all the included studies and relevant systematic reviews to identify additional potential studies for inclusion. The details of the search strategies for each database are provided in Multimedia Appendix 1.

### Study Eligibility Criteria

#### Overview

The study eligibility criteria were defined using the following PICOS (participants, interventions, comparisons, outcomes, and study designs or settings) framework. A study was included if it met all the following criteria.

#### Participants

We included the study if it was conducted on healthy pregnant women aged 15 to 49 years. If the study included a high-risk population, such as pregnant women with HIV/AIDS, cancer, preeclampsia, or other severe diseases at baseline, it was excluded because of higher medical adherence before intervention implementation among these groups. We considered low- or average-risk pregnant women, not high-risk pregnant women, as healthy pregnant women.

#### Interventions

We included all types of mHealth interventions that focused on improving perinatal health care utilization, including SBA and facility delivery.

#### Comparisons

We included studies that compared the effectiveness of any form of mHealth intervention (eg, voice calling, SMS text messaging, mobile apps, and videos) with standard care.

#### Outcomes

We included studies that reported ANC visits and skilled delivery care utilization, such as facility delivery and SBA during delivery.

#### Study Designs

We considered only RCTs and cluster RCTs in this review. Qualitative studies, case studies, cross-sectional studies, quasi-RCTs, quasi-experimental studies (controlled before and after studies), review studies, discussion papers, case reports, commentaries, editorials, expert opinions, and ongoing research with insufficient PICOS information were excluded.

#### Settings

We included studies conducted in LMICs based on the World Bank categories at the time of study implementation [25].

### Study Selection Process

Two reviewers (MOR and NY) independently screened the titles and abstracts of all retrieved studies and identified potentially relevant studies using the predefined study eligibility criteria. To assess their eligibility in detail, they independently critiqued all potentially relevant studies during the full-text screening stage. In both stages, disagreements were resolved through discussion or by a third reviewer (YN or EO), when required. We recorded the reasons for exclusion of all studies in the full-text screening stage and reported them in a PRISMA study flow diagram. We used EndNote (Niles Software) reference management software and the Rayyan Qatar Computing Research Institute tool in the study selection process [26].

### Study Quality Assessment

Two reviewers (MOR and NY) independently assessed the risk of bias of the included studies using the Cochrane risk of bias tool. The tool consists of the following domains: random sequence generation, allocation concealment, blinding of participants and personnel, blinding of outcome assessment, incomplete outcome data, selective outcome reporting, and other bias. For the domain of blinding of participants and personnel, we considered blinding of personnel only as blinding, because the nature of mHealth interventions, may not be possible to study participants. We classified the studies with high, low, and unclear risks of bias based on the Cochrane Handbook [27]. Any discrepancies were solved through discussion or by a third reviewer (YN or EO).

### Certainty of Evidence Assessment

We evaluated certainty of evidence for ANC, SBA, and facility delivery outcome using the Grading of Recommendation, Assessment, Development and Evaluation (GRADE) system [28]. The GRADE system considered the judgment on the following factors while assessing the confidence in the evidence based on RCT studies: study limitations (risk of bias), inconsistency (statistical heterogeneity), indirectness (PICO [participants, interventions, comparisons, and outcomes] and applicability), imprecision (number of events and CIs), and publication bias. On the basis of the judgment of each factor, we classified our evidence as follows: (1) high-certainty evidence (further research is very unlikely to change the confidence of the pooled results), (2) moderate-certainty evidence (further research is likely to have an important impact on the confidence of the pooled results and may change the estimate), (3) low-certainty evidence (further research is extremely likely to have an important impact on the confidence of the pooled results and likely to change the estimate), and (4) very low-certainty evidence (the pooled results have extreme uncertainty) [28]. We used the GRADEpro web-based platform to make a summary of findings table, considering the certainty of evidence.

### Data Extraction

Two independent reviewers (MOR and NY) extracted a standard set of data, including study characteristics, participant characteristics, description of interventions, and outcome results from each of the selected studies and were cross-checked. As in the study selection process, disagreements were resolved through discussion or by a third reviewer (YN or EO). We reported key characteristics of the included studies in a separate table. The data characteristics included, but were not limited to, author information, year of publication, study location, study setting, study design, study name, number of participants, study year, age of participants, gestational age at recruitment, comparator, types, function, mode and duration of interventions, intervention provider, and reported outcomes with their results.

### Data Synthesis and Analysis

We narratively synthesized study characteristics, participant characteristics, intervention characteristics and key findings among all included studies. To summarize the effect size of mHealth interventions, we used pairwise inverse-variance random-effects meta-analysis separately for each outcome. While pooling the effect size, we used risk ratios (RRs) because our outcome was dichotomous in nature. If the study provided odds ratios (ORs) and the risk of events in the control group (assumed control risk [ACR]), we converted ORs into RRs by using the formula (OR/[1 – ACR × (1 − OR)]) described in the Cochrane Handbook for Systematic Reviews of Intervention [29]. The results of the meta-analysis are presented in forest plots. In the meta-analysis, we used the estimated effective samples for cluster RCT studies by adjusting their design effect if the studies reported unadjusted data. Heterogeneity was assessed by visual inspection of forest plots or tested using the *I*^2^ statistic, and we considered an *I*^2^ value >50% to indicate substantial heterogeneity [29]. If substantial heterogeneity was found in the meta-analyses, we conducted a subgroup analysis based on the direction of interventions (1-way vs 2-way communication) and high baseline coverage of outcomes (80% or more vs <80%) and reported the subgroup-wise pooled estimates for each outcome separately. We used funnel plots and the Egger test to assess publication bias if a meta-analysis includes 10 or more studies. Statistical significance was defined as a *P* value <.05 for all analyses.

### Ethics Approval and Consent to Participate

This study did not require ethical approval or consent to participate, as it used data from published studies.

## Results

### Study Inclusion

**Figure 1:**
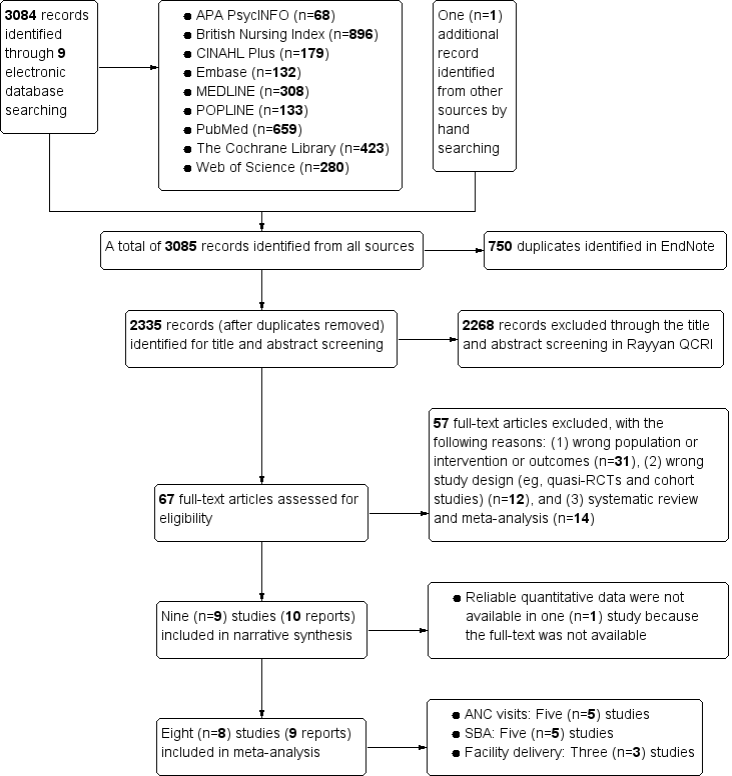
PRISMA study flow diagram. ANC: antenatal care; PRISMA: Preferred Reporting Item for Systematic Reviews and Meta-Analyses; QRCI: Qatar Computing Research Institute; RCT: randomized controlled trial; SBA: skilled birth attendance.

A total of 3085 potentially relevant articles were retrieved from all targeted electronic databases and other resources. After removing duplicates in EndNote, 2335 unique articles underwent initial title and abstract screening. As a result, 67 articles were retained for a detailed assessment of study eligibility. After full-text screening, 57 articles that failed to meet the study eligibility criteria were excluded. The reasons for exclusion are reported in the PRISMA study flow diagram (Figure 1). Finally, 10 articles (9 studies) from all resources were found to be suitable for narrative synthesis. We identified 1 gray article by checking the reference lists of all included studies and relevant systematic reviews, but the full text was not available [30]. As we could not check its quantitative information, we narratively synthesized its results and excluded it from our meta-analysis.

Of all 9 included studies, 6 (67%) assessed the effect of mHealth interventions on improving the uptake of ANC visits [17,30-34], 5 (56%) on SBA during delivery [16,31-33,35], and 3 (33%) on facility delivery outcome [31,36,37]. We performed a meta-analysis for ANC visits and SBA outcomes based on the direction of interventions (1-way or 2-way communication) and for facility delivery based on high coverage of outcomes at baseline (80% or more; Figure 1).

### Characteristics of the Included Studies

Of all 9 included studies, 6 (67%) were individual RCTs, and 3 (33%) were cluster RCTs (Table 1). Studies were conducted in Brazil [34], China [30], Ethiopia [31], India [32,35], Kenya [33,37], Nigeria [36], and Tanzania [16,17] and were published between 2012 and 2018. A total of 10,348 pregnant women (n=6254, 60.44% in the intervention groups and n=4094, 39.56% in the control groups) participated in the studies. The number of study participants ranged from 116 to 2160 in the intervention groups and from 100 to 1239 in the control groups.

Interventions were carried out through SMS text messaging (5/9, 56% studies), voice calling (1/9, 11% studies), SMS text messaging and mobile voucher (1/9, 11% studies), voice calling and SMS text messaging (1/9, 11% studies), and voice messaging and animation film clips (1/9, 11% studies). Approximately half of the studies used interventions for 1-way communication (4/9, 44% studies) or 2-way communications (5/9, 56% studies). The functions of interventions were categorized into appointment reminder (1/9, 11% studies), health education or advice and appointment reminder (5/9, 56% studies), and health education or preventive health information (3/9, 33% studies). The detailed characteristics of the interventions and the results of the included studies are presented in Table 2.

**Table 1:** Characteristics of included studies.

Characteristics		Studies, n (%)
**Study design (n=9)**		
	Individual RCTs^a^	6 (67)
	Cluster RCTs	3 (33)
**Countries (n=9)**		
	Brazil	1 (11)
	China	1 (11)
	Ethiopia	1 (11)
	India	2 (22)
	Kenya	2 (22)
	Nigeria	1 (11)
	Tanzania	1 (11)
**Publication year (n=9)**		
	2012	1 (11)
	2013	1 (11)
	2014	1 (11)
	2015	1 (11)
	2017	3 (33)
	2018	2 (22)
**Outcomes reported (n=9)**		
	4 or more ANC^b^	6 (67)
	SBA^c^	5 (56)
	Facility delivery	3 (33)
**Participants (excluded 1 study; n=10,348)**		
	Intervention group	6254 (60.44)
	Control group	4094 (39.56)
**Medium of interventions (n=9)**		
	SMS text messaging	5 (56)
	SMS text messaging and mobile voucher	1 (11)
	Voice calling	1 (11)
	Voice calling and SMS text messaging	1 (11)
	Voice messaging and animation film clips	1 (11)
**Direction of interventions (n=9)**		
	1-way communication	4 (44)
	2-way communication	5 (56)
**Function of interventions (n=9)**		
	Appointment reminder	1 (11)
	Health education or advice and appointment reminder	5 (56)
	Health education or preventive health information	3 (33)

^a^RCT: randomized controlled trial.

^b^ANC: antenatal care.

^c^SBA: skilled birth attendance.

**Table 2:** Characteristics of interventions and results of the included studies.

Authors	Country, participants, study design, sample size	Form of mHealth^a^ interventions (medium, direction, and function)	Control group	Reported outcomes	Key findings
Lund et al, 2012 [16]	Tanzania; pregnant women; cluster RCT^b^; intervention: n=1311; control: n=1239	Mobile phone SMS text messaging (twice a week) and mobile voucher; 2-way communication; health education and appointment reminder	Routine ANC^c^ and advice	SBA^d^ at delivery	Significantly increased skilled delivery attendance among pregnant women (OR^e^ 1.69, 95% CI 1.44-1.98)
Luo, 2013 [30]	China; pregnant women; individual RCT; intervention: not available; control: not available	Health education intervention through mobile phone SMS text messaging; 1-way communication	Usual care	4 or more ANC visits	Showed positive effect of health education intervention through mobile phone SMS text messaging on 4 or more ANC visits
Fedha, 2014 [33]	Kenya; pregnant women; individual RCT; intervention: n=191; control: n=206	Mobile phone reminder, updates, and advice: every fortnightly of the next visit to the clinic and given advice and updates on pregnancy; 2-way communication	Routine care with no mobile advice or update support	4 or more ANC visits, SBA, other birth outcomes	Mobile phone services for pregnant women enhanced 4 or more ANC visits (OR: 2.89, 95% CI 1.51-5.53) and SBA (OR 2.73, 95% CI 1.60-4.65)
Lund et al, 2014 [17]	Tanzania; pregnant women; cluster RCT; intervention: n=1311; control: n=1239	Mobile phone SMS text messaging (twice a week) and mobile voucher; 2-way communication; health education and appointment reminder	Routine ANC and advice	4 or more ANC visits; tetanus vaccination and other preventive services	44% of the women received 4 or more ANC visits in the intervention group versus 31% in the control group
Joshi et al, 2015 [35]	India; pregnant women; individual RCT (where most women already use a skilled birth attendant); intervention: n=1162; control: n=581	Preventive health information via voice messages and animation film clips (the automated platform for voice messages); 1-way communication	Usual care (no voice messages and animation films)	SBA, iron and folic acid tablet intake, and knowledge on ANC and their satisfaction	mHealth initiative for promoting higher uptake of ANC services is highly impactful
Atnafu et al, 2017 [31]	Ethiopia; pregnant women; a community-based RCT; intervention: n=1080 (group T1), n=1080 (group T2); control: n=1080	SMS text messaging–based mobile phone reminder intervention; 1-way communication	No SMS text messaging	Role of mobile phone SMS text messaging on MCH^f^ outcomes: 4 or more ANC visits, SBA, and facility delivery	Confirmed the positive contribution of SMS text messaging–based mobile phone intervention to most of the selected MCH service indicators, such as improvement in the percentage of recommended number of ANC visit and percentage of delivery attended by health workers
Bangal et al, 2017 [32]	India; pregnant women; individual RCT; intervention: n=200; control: n=200	Mobile phone calls, as reminders about next visit, and SMS text messaging on important aspects of ANC at regular intervals; 1-way communication	Routine ANC and advice as per hospital protocol	Percentage of pregnant women coming for at least four ANC visits and percentage of institutional delivery and postnatal checkups	Women in the intervention group had significantly higher number of ANC visits, consumption of iron tablets, tetanus toxoid immunization, institutional deliveries and postnatal checkups as compared with the control group
Oliveira-Ciabati et al, 2017 [34]	Brazil; pregnant women; cluster RCT; intervention: n=770 (PRENACEL group: n=116); control: n=440	PRENACEL group received a weekly set of SMS text messages with health education and health promotion content related to pregnancy and childbirth and were also able to clarify ANC queries through SMS text messaging; 2-way communication	Routine ANC	ANC, tetanus vaccination, influenza vaccination, and other preventive services	A bidirectional, mobile phone–based, SMS text messaging service is potentially useful for improving the coverage of recommended ANC practices, including syphilis and HIV testing
Omole et al, 2018 [36]	Nigeria; pregnant women; cluster RCT; intervention: n=260; control: n=248	Pregnant women in the intervention facilities received pregnancy‐related health messages and reminders for their ANC appointments through SMS text messaging and also had the opportunity of sending SMS text messages to the project team to seek for health information; 2-way communication	Only received general health messages through SMS text messaging	Attendance of at least four ANC clinic visits and delivery in a health facility	Most of the pregnant women in the intervention group (96.6%) expressed support for the use of SMS text messaging for maternal health promotion. The SMS text messaging–based intervention has a positive effect on facility delivery. A 13% increase was recorded in the rate of facility‐based delivery among the control group between the last and index degrees, a much higher 29% increase was recorded among the intervention group
Unger et al, 2018 [37]	Kenya; pregnant women; individual RCT; intervention: n=200 (n=100, 1-way; n=100, 2-way); control: n=100	An automated weekly gestational age-appropriate educational and counseling messaging, and SMS text messaging topic included ANC, pregnancy complications, family planning, infant health, EBF^g^, infant immunization, and visit reminders; 1-and 2-way communications	Routine clinic–based counseling and care	Facility delivery, EBF, and contraceptive use	Facility delivery was very high in all 3 arms (98.6%). The mobile WACh SMS text messaging intervention had no effect on the uptake of facility delivery

^a^mHealth: mobile health.

^b^RCT: randomized controlled trial.

^c^ANC: antenatal care.

^d^SBA: skilled birth attendance.

^e^OR: odds ratio.

^f^MCH: maternal and child health.

^g^EBF: exclusive breastfeeding.

### Overall Risk of Bias Assessment of the Included Studies

**Figure 2:**
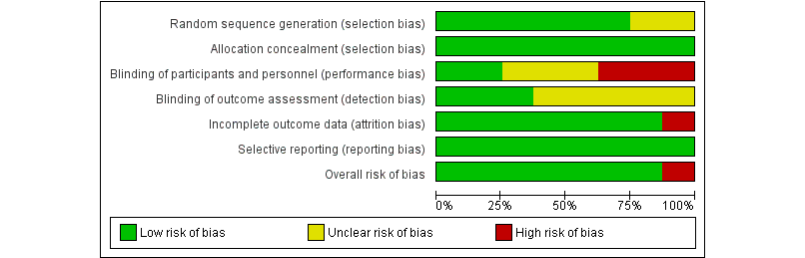
Risk of bias graph: review of authors’ judgments about each risk of bias item presented as percentages across all included studies.

**Figure 3:**
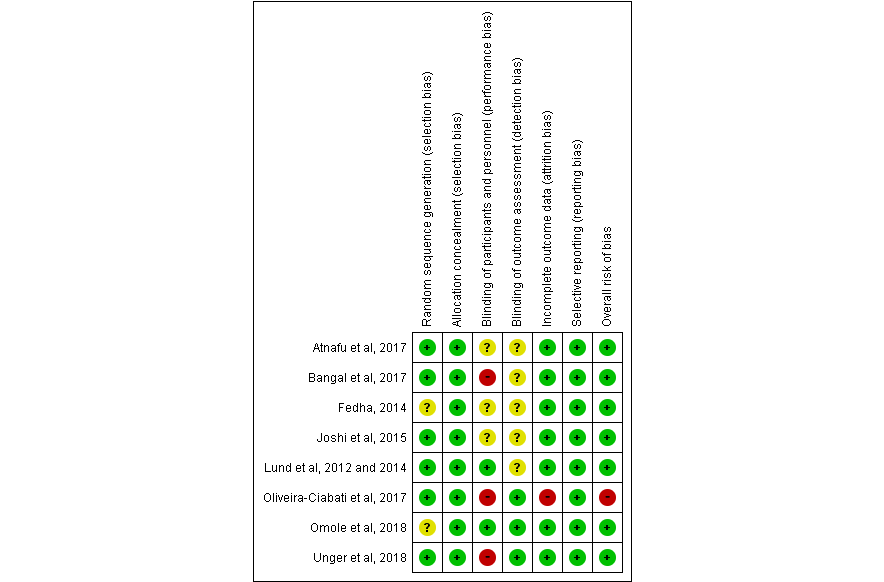
Risk of bias summary: review of authors’ judgments about each risk of bias item for each included study [17,31-37].

A summary of the overall risk of bias assessment is presented in Figure 2 and Figure 3. RCT studies generally performed well in their risk of bias for random sequence generation (75% low risk, with the remainder unclear), allocation concealment (100% low risk), blinding outcome assessment (37.5% low risk, with the remainder unclear), incomplete outcome data (87.5% low risk), and selective reporting (100% low risk). A total of 3 studies [32,34,37] had a high risk of bias regarding blinding participants and personnel, and another study [34] also had a high risk of bias for incomplete outcome data. In addition, 1 study [33] had an unclear risk for many items. Overall, most of the studies had a low risk of bias.

### Pooled Effects of mHealth Interventions on the Uptake of ANC Utilization

**Figure 4:**
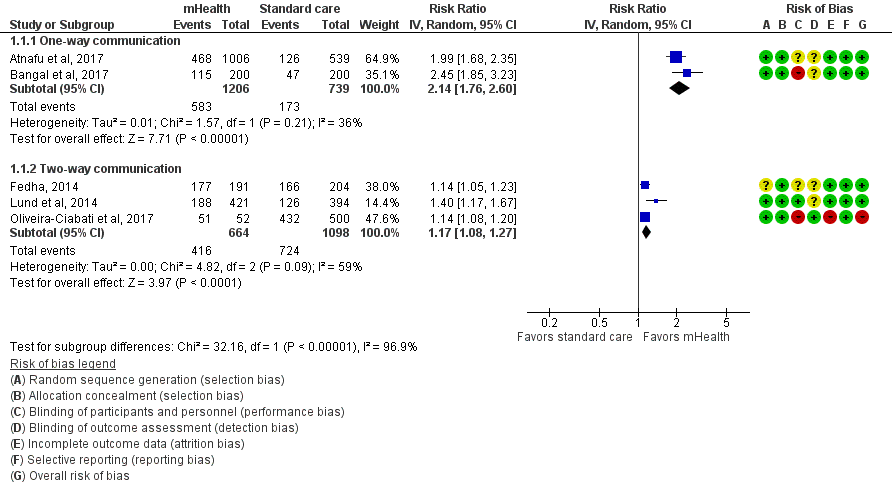
Meta-analysis for the effect of mHealth interventions versus standard care on 4 or more ANC visits among pregnant women. ANC: antenatal care; mHealth: mobile health.

We performed an inverse-variance random-effects meta-analysis to summarize the effects of 1-way and 2-way mHealth interventions versus standard care on the uptake of 4 or more ANC visits among pregnant women (Figure 4 and Table 3). A total of 2 studies [31,32] consisting of 1945 pregnant women (n=1206, 62.01% in the intervention group and n=739, 37.99% in the control group) implemented a 1-way mHealth intervention and were included in the meta-analysis. The pooled estimates showed a significantly large risk with a 114% increase in 4 or more ANC visits among pregnant women given a 1-way mHealth intervention, compared with the control group (RR 2.14, 95% CI 1.76-2.60, *I*^2^=36%, moderate certainty of evidence).

In total, 3 studies [17,33,34] consisting of 1762 pregnant women (n=664, 37.68% in the intervention group and n=1098, 62.32% in the control group) reported 2-way mHealth intervention, and the pooled estimates from the meta-analysis showed that the risk of 4 or more ANC visits was 17% higher in the 2-way mHealth intervention group than in the control group (RR 1.17, 95% CI 1.08-1.27, *I*^2^=59%, low certainty of evidence). Although all studies reported a positive effect of mHealth interventions, regardless of the direction of interventions, on improving the uptake of ANC visit utilization [17,31-34], we observed a significant difference between these subgroups (*I*^2^=96.9%), which limited their combination.

**Table 3:** Summary of findings.^a^

Outcomes	Anticipated absolute effects^b^		Relative effect, RR^c^ (95% CI)	Number of participants (studies)	Certainty of the evidence (GRADE^d,e^)	Comments
	Risk with standard care	Risk with mHealth^f^ intervention (95% CI)				
4 or more ANC^g^ visits (1-way communication)	234 per 1000	501 per 1000 (412-609)	2.14 (1.76-2.60)	1945 (2 RCTs^h^)	Moderate^i^	One-way mHealth intervention likely results in large increase in 4 or more ANC visit utilizations among pregnant women in LMICs^j^, and further research may change the estimate.
4 or more ANC visits (2-way communication)	659 per 1000	771 per 1000 (712-837)	1.17 (1.08-1.27)	1762 (3 RCTs)	Low^k,l^	Two-way mHealth intervention may result in an increase in 4 or more ANC visit utilizations among pregnant women in LIMCs and further research is likely to change the estimate.
SBA^m^ (1-way communication)	771 per 1000	802 per 1000 (748-848)	1.04 (0.97-1.10)	3460 (3 RCTs)	Very low^i,l,n^	One-way mHealth intervention may not increase SBA during delivery in LMICs, but the evidence is very uncertain.
SBA (2-way communication)	557 per 1000	685 per 1000 (635-740)	1.23 (1.14-1.33)	1212 (2 RCTs)	Moderate^o^	Two-way mHealth intervention likely results in an increase in SBA during delivery among pregnant women in LMICs, and further research may change the estimate.
Facility delivery (<80% at baseline)	360 per 1000	604 per 1000 (467-787)	1.68 (1.30-2.19)	1819 (2 RCTs)	Moderate^o^	mHealth intervention likely results in an increase in facility delivery in LMICs where fewer pregnant women use facility delivery, and further research may change the estimate.
Facility delivery (80% or more at baseline)	990 per 1000	1000 per 1000 (960-1000)	1.01 (0.97-1.04)	300 (1 RCT)	Low^n,p^	mHealth intervention may not increase facility delivery in LMICs where most pregnant women already use facility delivery, and further research is likely to change the estimate.

^a^mHealth intervention compared with standard care for improving ANC utilization, SBA during delivery, and facility delivery among pregnant women. Population: pregnant women; setting: LMICs (Brazil, China, Ethiopia, India, Kenya, Nigeria, and Tanzania); intervention: mHealth intervention; comparison: standard care.

^b^The risk in the intervention group (and its 95% CI) is based on the assumed risk in the comparison group and the relative effect of the intervention (and its 95% CI).

^c^RR: risk ratio.

^d^GRADE: Grading of Recommendation, Assessment, Development and Evaluation.

^e^The GRADE Working Group grades of evidence. High certainty: we are very confident that the true effect lies close to that of the estimate of the effect. Moderate certainty: we are moderately confident in the effect estimate—the true effect is likely to be close to the estimate of the effect, but there is a possibility that it is substantially different. Low certainty: our confidence in the effect estimate is limited—the true effect may be substantially different from the estimate of the effect. Very low certainty: we have very little confidence in the effect estimate—the true effect is likely to be substantially different from the estimate of effect.

^f^mHealth: mobile health.

^g^ANC: antenatal care.

^h^RCT: randomized controlled trial.

^i^Unclear or lack of blinding of participants and outcome assessors.

^j^LMICs: low- and middle-income countries.

^k^Unclear or lack of sequence generation, blinding of participants and outcome assessors, and incomplete outcome data.

^l^Statistical heterogeneity (*I*^2^>50%).

^m^SBA: skilled birth attendance.

^n^CI crossed the threshold.

^o^Unclear sequence generation, blinding of participants, and outcome assessors.

^p^Lack of blinding of participants and personnel.

### Pooled Effects of mHealth Interventions on SBA at the Time of Delivery

**Figure 5:**
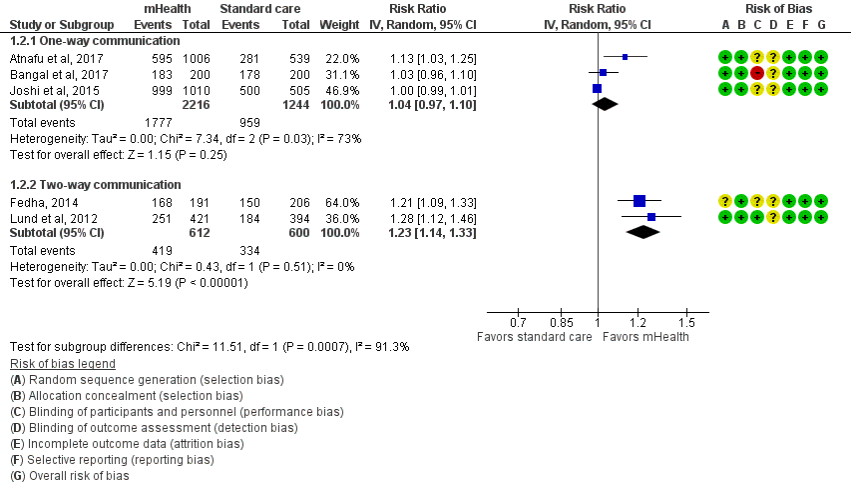
Meta-analysis for the effect of mHealth interventions versus standard care on SBA during delivery. mHealth: mobile health; SBA: skilled birth attendance.

An inverse-variance random-effects meta-analysis was performed to pool the effects of 1-way and 2-way mHealth interventions versus standard care on improving the rate of SBA at the time of delivery (Figure 5 and Table 3). In total, 3 studies [31,32,35] comprising 3460 pregnant women (n=2216, 64.05% in the intervention group and n=1244, 35.85% in the control group) reported a 1-way mHealth intervention in which only 1 study [31] found a positive effect of the intervention on SBA during delivery. The effects of 1-way mHealth interventions on SBA during delivery were not clear; however, the effects were pooled in the meta-analysis (RR 1.04, 95% CI 0.97-1.10, *I*^2^=73%, very low certainty of evidence).

In total, 2 studies [16,33] involving 1212 pregnant women (n=612, 50.50% in the intervention group and n=600, 49.50% in the control group) implemented 2-way mHealth interventions, and their pooled estimates showed that the proportion of SBA during delivery was 23% higher in the 2-way mHealth intervention group than in the control group (RR 1.23, 95% CI 1.14-1.33, *I*^2^=0%, moderate certainty of evidence). Owing to significant subgroup differences in the direction of mHealth interventions (*I*^2^=91.3%), we could not combine their effects.

### Pooled Effects of mHealth Interventions on Facility Delivery Among Pregnant Women

**Figure 6:**
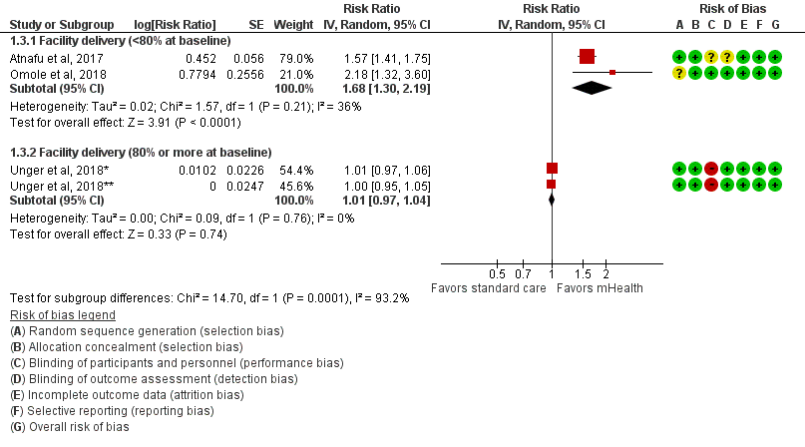
Meta-analysis for the effect of mHealth interventions versus standard care on facility delivery among pregnant women. mHealth: mobile health; *1-way mHealth intervention; **2-way mHealth intervention.

Overall, 3 studies assessed the effects of mHealth interventions on facility delivery outcome [31,36,37]. In addition, 1 study [31] found that the probability of facility delivery was 57% higher in the 1-way mHealth intervention group than in the control group (RR 1.57, 95% CI 1.41-1.75). Another study [36] identified a positive result supporting the effectiveness of 2-way mHealth interventions for improving facility delivery (RR 2.18, 95% CI 1.32-3.60). However, 1 study reported an unclear effect of the mHealth interventions, irrespective of direction, on the uptake of facility delivery [37]. We conducted a subgroup analysis considering high coverage of facility delivery at baseline (80% or more) and found a positive effect of mHealth interventions on improving the uptake of facility delivery in LMICs, where fewer pregnant women used facility delivery at baseline (RR 1.68, 95% CI 1.30-2.19, *I*^2^=36%, moderate certainty of evidence); however, the effects were unclear where most pregnant women already used facility delivery (RR 1.01, 95% CI 0.97-1.04, *I*^2^=0%, low certainty of evidence). Significant subgroup differences (*I*^2^=93.2%) limited the combination of these effects (Figure 6 and Table 3).

### Subgroup Differences

Significant subgroup differences (*P*<.001) were found in all meta-analyses, which limited the combination of the effects of all included studies (Figures 4-6). It included an estimated 96.9% subgroup variance for 4 or more ANC visits, 91.3% for SBA at the time of delivery, and 93.2% for facility delivery.

### Publication Bias

Although we planned to assess the publication bias of our meta-analyses, we did not perform the analysis because of the limited number of studies.

## Discussion

### Principal Findings

This meta-analysis identified a statistically significant positive effect of mHealth interventions, regardless of the direction of interventions (1-way or 2-way communications), on improving ANC care utilization of healthy pregnant women in LMICs. Only 2-way mHealth interventions were effective in improving SBA during delivery, but the effects were unclear for 1-way mHealth interventions compared with standard care. For facility delivery among healthy pregnant women, the interventions were effective in settings where fewer pregnant women used facility delivery. Most studies that combined multiple mHealth interventions were implemented in Brazil, China, Ethiopia, India, Kenya, Nigeria, and Tanzania. The functions or directions of interventions varied among the included studies, such as 1-way or 2-way communication, appointment reminder, or health advice. All studies, except one [34], had low concerns of their methodological qualities. However, high statistical heterogeneity limited the combination of subgroups in all the meta-analyses.

These findings are consistent with a systematic review reporting that mHealth interventions had a positive effect and resulted in a 43% increase in the uptake of recommended ANC visits among pregnant women [13]. In another systematic review conducted by Wagnew et al [19], SMS text messaging had positive effects on the uptake of 4 or more ANC visits (OR 2.74, 95% CI 1.41-5.32) and SBA (OR 1.82, 95% CI 1.33-2.49) in LMICs [19], which strongly supports our findings. Although the availability of high-level evidence such as RCTs on mHealth interventions targeting healthy pregnant women’s health care utilization is still very limited, our findings have generated promising results regarding the positive effects of mHealth interventions, regardless of their directions, on improving recommended ANC utilization, SBA during delivery, and facility delivery in resource-limited countries. The findings are also consistent with other systematic reviews that found that mHealth tools are effective in influencing maternal and child health service utilization by enhancing the uptake of recommended ANC and postnatal care services, including SBA, at the time of delivery and institutional delivery [12,14,18].

The effects of 1-way mHealth interventions on SBA at the time of delivery were not clear in the 2 included studies [32,35], which was reflected in our pooled results. Our meta-analysis identified a significant difference in the likelihood of SBA during delivery, which was higher in the 2-way mHealth intervention group than in the control group. Our findings support the effectiveness of mHealth interventions in improving facility delivery in settings where fewer pregnant women use the service. Consistent with our findings, a systematic review conducted by Colaci et al [38] reported that mHealth interventions offered an opportunity to increase the acceptability of prenatal and obstetric care, including SBA at the time of delivery. This is because mHealth interventions, either health care reminders or health advice, boost self-efficacy and access to care among pregnant women and create closer interactions with health service providers. Furthermore, mHealth interventions have been used as appointment reminders and can provide basic health information, notably throughout the pregnancy period. As novel and more cost-effective systems are being sought to promote health care utilization in underserved areas, particularly in remote and rural areas, this intervention offers a potential cost-effective solution. This study strongly supports the use of mHealth interventions, either 1-way or 2-way communication, to enhance the uptake of maternal health care services such as ANC, SBA, or facility delivery, by changing health care behavior among pregnant women in similar settings.

### Strengths

This review had several strengths. To the best of our knowledge, this is the first comprehensive review that conducted meta-analyses for different subgroups, even in similar settings, for each outcome, such as the direction of interventions (1-way or 2-way communication) and high coverage of outcomes at baseline, and identified some novel findings not seen in previous studies. For example, 2-way (not 1-way) mHealth interventions likely result in an increase in SBA during delivery in resource-limited settings. Likewise, mHealth interventions likely result in an increase in facility delivery in LMICs where fewer pregnant women use the service but may not increase in settings where most pregnant women already use facility delivery. Interestingly, the certainty of the evidence was moderate, indicating that our estimates were likely to be close to the true effect.

Second, we conducted a comprehensive search of electronic databases without any limit on language, date, or type of publication. We also checked the reference lists of the included studies and other relevant systematic reviews to avoid missing any potentially relevant studies, and identified 1 additional study. Third, this study considered high-level studies such as RCTs and cluster RCTs and performed meta-analyses well. In the meta-analyses, we used the estimated effective samples for cluster RCT studies; however, the studies did not adjust their design effect. In addition, with the larger sample size, we enhanced the statistical power to provide more precise and reliable effect estimates.

### Limitations

Despite the positive effects of mHealth interventions reported in our meta-analyses, this review had several limitations. One of the important limitations is the limited number of RCT studies included in the meta-analyses (8 studies). All included studies were reported from only 7 LMICs, and some used small sample sizes that may compromise representativeness. In addition, approximately half of the studies tried to combine multiple mHealth interventions, making it difficult to understand the extent to which each intervention contributed to the observed results. For instance, a study assessed the effectiveness of a combined intervention of mobile SMS text messaging and mobile voucher [16,17], another studied a voice messaging and animation film clip intervention [35] and a mobile phone call and SMS text messaging intervention [32]. Likewise, some studies tried to combine multiple functions of mHealth interventions (eg, health education or advice and appointment reminder), which made it difficult to determine the extent to which each function of intervention contributed to the reported outcomes [16,17,32,33,36].

Although the mHealth interventions were effective in improving facility delivery in the settings where fewer pregnant women used the service, we need to interpret these results with caution because it is not clear if the benefits presented regarding facility delivery are a function of the mHealth interventions or greater health literacy and knowledge of navigating the local health care system among users and because it is also not clear if the increase in ANC and SBA are due to the mHealth technology or access to health care. With the mHealth interventions, we also need to consider other factors such as sociocultural norms and beliefs during perinatal periods, perinatal care availability and resources, or other structural factors to improve ANC, SBA, and facility delivery utilization among pregnant women.

We performed an inverse-variance random-effects meta-analysis to summarize the effect of mHealth interventions, irrespective of their directions or high coverage of outcomes at baseline, on the uptake of ANC visits, SBA, and facility delivery utilization among pregnant women in LMICs; however, we observed a significant difference between these subgroups that limited their combination. Within the subgroups, we did not observe considerable heterogeneity among the included studies.

### Implications for Future Research

mHealth interventions (1-way or 2-way communication) may contribute toward improving maternal health care–seeking behavior throughout the pregnancy cycle. Public health researchers, practitioners, and policy makers should consider such interventions in resource-limited settings. It is reasonable to use mHealth interventions in resource-limited settings, as this study found a positive effect of different forms of intervention on the uptake of ANC visits, SBA, and facility delivery utilization. This study could play an important role in addressing the Sustainable Development Goal of Maternal and Child Health, as it provides insights and evidence-informed recommendations for the utilization of different forms of mHealth interventions in addressing maternal health care challenges in LMICs. However, owing to the limited number of RCT studies that met the study eligibility criteria in this review, further large-scale RCTs are suggested to be implemented in resource-limited countries, particularly where service utilization is quite low among pregnant women. In recent times, most LMICs have recognized the need for appropriate technology use strategies to promote the utilization of their existing health systems, which also justifies the necessity of further evidence of technology-based health care interventions such as mHealth interventions.

### Conclusions

Although mHealth interventions (1-way or 2-way communication) can improve the uptake of ANC and skilled delivery care utilization, more rigorous evaluations are required to strengthen the evidence of different forms of mHealth interventions for improving the existing health care utilization among healthy pregnant women in LMICs. This systematic review and meta-analysis will help public health researchers or policy makers in designing and implementing mHealth interventions in resource-limited settings, as this study identified some novel findings not found in previous reviews.

## Appendix

Multimedia Appendix 1 Search strategy of electronic databases.

## Abbreviations

ANC: antenatal care
GRADE: Grading of Recommendation, Assessment, Development and Evaluation
LMIC: low- and middle-income country
mHealth: mobile health
OR: odds ratio
PICO: participants, interventions, comparisons, and outcomes
PICOS: participants, interventions, comparisons, outcomes, and study designs or settings
PRISMA: Preferred Reporting Items for Systematic Reviews and Meta-Analyses
RCT: randomized controlled trial
RR: risk ratio
SBA: skilled birth attendance

## Data Availability

No additional data available.
