# Supplementary material for: Effects of mHealth Interventions on Improving Antenatal Care Visits and Skilled Delivery Care in Low- and Middle-Income Countries: Systematic Review and Meta-analysis
Source: J Med Internet Res. 2022 Apr 22;24(4):e34061. doi: 10.2196/34061 (PMC9077501; doi:10.2196/34061)
Supplement: Multimedia Appendix 1 [file jmir_v24i4e34061_app1.docx]

Multimedia Appendix 1: Electronic database search strategy

**Date of search: October 2020**

**Search history:**

| **SL** | **Database** | **# Hits** |
| --- | --- | --- |
|  | APA PsycINFO | 68 |
|  | British Nursing Index | 896 |
|  | CINAHL PLUS | 179 |
|  | EMBASE | 132 |
|  | MEDLINE | 308 |
|  | POPLINE | 133 |
|  | PubMed | 659 |
|  | The Cochrane Library | 424 |
|  | Web of Science | 280 |
| **Total** | | **3,084** |
| **Duplicates** | | 750 |
| **Total for screening (after duplicate removed)** | | **2,334** |

**Search history of APA PsycINFO:**

| **SL** | **Query** | **# Hits** |
| --- | --- | --- |
|  | TI (expectant mother OR pregnant women OR pregnant mother OR pregnancy) OR AB (expectant mother OR pregnant women OR pregnant mother OR pregnancy) | 39,411 |
|  | TI (computer OR tablet OR phone OR mobile OR mobile phone OR mobile device OR smartphone OR smart-phone OR cell phone OR cellphone OR cellular phone OR web OR website OR Internet OR online OR on-line OR technology OR digital technology OR mobile technology OR health technology OR wireless technology OR wireless device OR iPhone OR i-Phone OR iPad OR i-Pad OR iPod OR i-Pod) OR AB (computer OR tablet OR phone OR mobile OR mobile phone OR mobile device OR smartphone OR smart-phone OR cell phone OR cellphone OR cellular phone OR web OR website OR Internet OR online OR on-line OR technology OR digital technology OR mobile technology OR health technology OR wireless technology OR wireless device OR iPhone OR i-Phone OR iPad OR i-Pad OR iPod OR i-Pod) | 344,224 |
|  | TI (SMS OR short message service OR short messaging OR mobile phone messaging OR MMS OR multimedia message service OR multi-media message OR SMS advice OR SMS reminder OR text message OR text messaging OR texting) OR AB (SMS OR short message service OR short messaging OR mobile phone messaging OR MMS OR multimedia message service OR multi-media message OR SMS advice OR SMS reminder OR text message OR text messaging OR texting) | 3,934 |
|  | TI (mobile call OR mobile calling OR mobile communication OR voice call OR voice calling OR voice message OR video conference) OR AB (mobile call OR mobile calling OR mobile communication OR voice call OR voice calling OR voice message OR video conference) | 1,049 |
|  | TI (mobile applications OR mobile apps OR mobile app OR smartphone app OR app OR apps OR email OR e-mail OR personal digital assistant OR PDA) OR AB (mobile applications OR mobile apps OR mobile app OR smartphone app OR app OR apps OR email OR e-mail OR personal digital assistant OR PDA) | 15,346 |
|  | S2 OR S3 OR S4 OR S5 | 352,922 |
|  | TI (eHealth OR e-Health OR electronic health OR digital health OR telehealth OR telemedicine OR telecommunication OR mHealth OR m-Health OR mobile health OR mobile medicine OR mcare OR m-care OR mobile care OR mHealth messaging OR mobile telehealth OR mobile telehealth care OR m-Edu OR medu OR m-education OR mobile education OR mLearning OR eLearning) OR AB (eHealth OR e-Health OR electronic health OR digital health OR telehealth OR telemedicine OR telecommunication OR mHealth OR m-Health OR mobile health OR mobile medicine OR mcare OR m-care OR mobile care OR mHealth messaging OR mobile telehealth OR mobile telehealth care OR m-Edu OR medu OR m-education OR mobile education OR mLearning OR eLearning) | 8,524 |
|  | S6 AND S7 | 5,163 |
|  | S1 AND S8 | 68 |

**Search history of BNI:**

| **SL** | **Query** | **# Hits** |
| --- | --- | --- |
|  | TI(expectant mother OR pregnant women OR pregnant mother OR pregnancy) OR AB(expectant mother OR pregnant women OR pregnant mother OR pregnancy) OR SU(expectant mother OR pregnant women OR pregnant mother OR pregnancy) | 64,988 |
|  | computer OR tablet OR phone OR mobile OR mobile phone OR mobile device OR smartphone OR smart-phone OR cell phone OR cellphone OR cellular phone OR web OR website OR Internet OR online OR on-line OR technology OR digital technology OR mobile technology OR health technology OR wireless technology OR wireless device OR iPhone OR i-Phone OR iPad OR i-Pad OR iPod OR i-Pod | 215,192 |
|  | SMS OR short message service OR short messaging OR mobile phone messaging OR MMS OR multimedia message service OR multi-media message OR SMS advice OR SMS reminder OR text message OR text messaging OR texting | 10,698 |
|  | mobile call OR mobile calling OR mobile communication OR voice call OR voice calling OR voice message OR video conference | 19,017 |
|  | mobile applications OR mobile apps OR mobile app OR smartphone app OR app OR apps OR email OR electronic mail OR e-mail OR personal digital assistant OR PDA | 59,367 |
|  | #2 OR #3 OR #4 OR #5 | 243,357 |
|  | eHealth OR e-Health OR electronic health OR digital health OR telehealth OR telemedicine OR telecommunication OR mHealth OR m-Health OR mobile health OR mobile medicine OR mcare OR m-care OR mobile care OR mHealth messaging OR mobile telehealth OR mobile telehealth care OR m-Edu OR medu OR m-education OR mobile education OR mLearning OR eLearning | 48,627 |
|  | #6 AND #7 | 41,070 |
|  | 'clinical trial'/de OR 'randomized controlled trial'/de OR 'randomization'/de OR 'single blind procedure'/de OR 'double blind procedure'/de OR 'crossover procedure'/de OR 'placebo'/de OR 'prospective study'/de OR 'randomi?ed controlled' NEXT/1 trial* OR rct OR 'randomly allocated' OR 'allocated randomly' OR 'random allocation' OR allocated NEAR/2 random OR single NEXT/1 blind* OR double NEXT/1 blind* OR (treble OR triple) NEAR/1 blind* OR placebo* | 30,901 |
|  | #1 AND #8 AND #9 | 896 |

**Search history of CINAHL PLUS:**

| **SL** | **Query** | **# Hits** |
| --- | --- | --- |
|  | TI (expectant mother OR pregnant women OR pregnant mother OR pregnancy) OR AB (expectant mother OR pregnant women OR pregnant mother OR pregnancy) | 99,147 |
|  | TI (computer OR tablet OR phone OR mobile OR mobile phone OR mobile device OR smartphone OR smart-phone OR cell phone OR cellphone OR cellular phone OR web OR website OR Internet OR online OR on-line OR technology OR digital technology OR mobile technology OR health technology OR wireless technology OR wireless device OR iPhone OR i-Phone OR iPad OR i-Pad OR iPod OR i-Pod) OR AB (computer OR tablet OR phone OR mobile OR mobile phone OR mobile device OR smartphone OR smart-phone OR cell phone OR cellphone OR cellular phone OR web OR website OR Internet OR online OR on-line OR technology OR digital technology OR mobile technology OR health technology OR wireless technology OR wireless device OR iPhone OR i-Phone OR iPad OR i-Pad OR iPod OR i-Pod) | 311,358 |
|  | TI (SMS OR short message service OR short messaging OR mobile phone messaging OR MMS OR multimedia message service OR multi-media message OR SMS advice OR SMS reminder OR text message OR text messaging OR texting) OR AB (SMS OR short message service OR short messaging OR mobile phone messaging OR MMS OR multimedia message service OR multi-media message OR SMS advice OR SMS reminder OR text message OR text messaging OR texting) | 3,622 |
|  | TI (mobile call OR mobile calling OR mobile communication OR voice call OR voice calling OR voice message OR video conference) OR AB (mobile call OR mobile calling OR mobile communication OR voice call OR voice calling OR voice message OR video conference) | 634 |
|  | TI (mobile applications OR mobile apps OR mobile app OR smartphone app OR app OR apps OR email OR e-mail OR personal digital assistant OR PDA) OR AB (mobile applications OR mobile apps OR mobile app OR smartphone app OR app OR apps OR email OR e-mail OR personal digital assistant OR PDA) | 16,126 |
|  | S2 OR S3 OR S4 OR S5 | 320,729 |
|  | TI (eHealth OR e-Health OR electronic health OR digital health OR telehealth OR telemedicine OR telecommunication OR mHealth OR m-Health OR mobile health OR mobile medicine OR mcare OR m-care OR mobile care OR mHealth messaging OR mobile telehealth OR mobile telehealth care OR m-Edu OR medu OR m-education OR mobile education OR mLearning OR eLearning) OR AB (eHealth OR e-Health OR electronic health OR digital health OR telehealth OR telemedicine OR telecommunication OR mHealth OR m-Health OR mobile health OR mobile medicine OR mcare OR m-care OR mobile care OR mHealth messaging OR mobile telehealth OR mobile telehealth care OR m-Edu OR medu OR m-education OR mobile education OR mLearning OR eLearning) | 22,243 |
|  | S6 AND S7 | 9,323 |
|  | S1 AND S8 | 179 |

**Search history of EMBASE:**

| **SL** | **Query** | **# Hits** |
| --- | --- | --- |
|  | 'expectant mother'/exp OR 'expectant mother' OR 'pregnant women'/exp OR 'pregnant women' OR 'pregnant mother' OR 'pregnancy'/exp OR pregnancy | 530,824 |
|  | 'computer'/exp OR computer OR 'tablet'/exp OR tablet OR phone OR mobile OR 'mobile phone'/exp OR 'mobile phone' OR 'mobile device'/exp OR 'mobile device' OR 'smartphone'/exp OR smartphone OR 'smart phone'/exp OR 'smart phone' OR 'cell phone'/exp OR 'cell phone' OR 'cellphone'/exp OR cellphone OR 'cellular phone'/exp OR 'cellular phone' OR 'web'/exp OR web OR 'website'/exp OR website OR 'internet'/exp OR internet OR 'online'/exp OR online OR 'on line' OR 'technology'/exp OR technology OR 'digital technology'/exp OR 'digital technology' OR 'mobile technology'/exp OR 'mobile technology' OR 'health technology'/exp OR 'health technology' OR 'wireless technology'/exp OR 'wireless technology' OR 'wireless device' OR 'iphone'/exp OR iphone OR 'i phone' OR 'ipad'/exp OR ipad OR 'i pad' OR 'ipod'/exp OR ipod OR 'i pod' | 1,075,331 |
|  | sms OR 'short message service'/exp OR 'short message service' OR 'short messaging' OR 'mobile phone messaging' OR mms OR 'multimedia message service' OR 'multi-media message' OR 'sms advice' OR 'sms reminder' OR 'text message'/exp OR 'text message' OR 'text messaging'/exp OR 'text messaging' OR 'texting'/exp OR texting | 15,996 |
|  | 'mobile call' OR 'mobile calling' OR 'mobile communication' OR 'voice call' OR 'voice calling' OR 'voice message' OR 'video conference'/exp OR 'video conference' | 898 |
|  | 'mobile applications'/exp OR 'mobile applications' OR 'mobile apps'/exp OR 'mobile apps' OR 'mobile app'/exp OR 'mobile app' OR 'smartphone app' OR app OR apps OR 'email'/exp OR email OR 'electronic mail'/exp OR 'electronic mail' OR 'e mail'/exp OR 'e mail' OR 'personal digital assistant'/exp OR 'personal digital assistant' OR pda | 71,991 |
|  | #2 OR #3 OR #4 OR #5 | 1,137,054 |
|  | 'ehealth'/exp OR ehealth OR 'e health'/exp OR 'e health' OR 'electronic health' OR 'digital health'/exp OR 'digital health' OR 'telehealth'/exp OR telehealth OR 'telemedicine'/exp OR telemedicine OR 'telecommunication'/exp OR telecommunication OR 'mhealth'/exp OR mhealth OR 'm health' OR 'mobile health'/exp OR 'mobile health' OR 'mobile medicine' OR mcare OR 'm care' OR 'mobile care' OR 'mhealth messaging' OR 'mobile telehealth' OR 'mobile telehealth care' OR 'm edu' OR medu OR 'm education' OR 'mobile education' OR mlearning OR elearning | 47,604 |
|  | #6 AND #7 | 21,001 |
|  | 'clinical trial'/de OR 'randomized controlled trial'/de OR 'randomization'/de OR 'single blind procedure'/de OR 'double blind procedure'/de OR 'crossover procedure'/de OR 'placebo'/de OR 'prospective study'/de OR 'randomi?ed controlled' NEXT/1 trial* OR rct OR 'randomly allocated' OR 'allocated randomly' OR 'random allocation' OR allocated NEAR/2 random OR single NEXT/1 blind* OR double NEXT/1 blind* OR (treble OR triple) NEAR/1 blind* OR placebo* | 2,106,610 |
|  | #1 AND #8 AND #9 | 132 |

**Search history of MEDLINE:**

| **SL** | **Query** | **# Hits** |
| --- | --- | --- |
|  | TI (expectant mother OR pregnant women OR pregnant mother OR pregnancy) OR AB (expectant mother OR pregnant women OR pregnant mother OR pregnancy) | 415,534 |
|  | TI (computer OR tablet OR phone OR mobile OR mobile phone OR mobile device OR smartphone OR smart-phone OR cell phone OR cellphone OR cellular phone OR web OR website OR Internet OR online OR on-line OR technology OR digital technology OR mobile technology OR health technology OR wireless technology OR wireless device OR iPhone OR i-Phone OR iPad OR i-Pad OR iPod OR i-Pod) OR AB (computer OR tablet OR phone OR mobile OR mobile phone OR mobile device OR smartphone OR smart-phone OR cell phone OR cellphone OR cellular phone OR web OR website OR Internet OR online OR on-line OR technology OR digital technology OR mobile technology OR health technology OR wireless technology OR wireless device OR iPhone OR i-Phone OR iPad OR i-Pad OR iPod OR i-Pod) | 1,722,556 |
|  | TI (SMS OR short message service OR short messaging OR mobile phone messaging OR MMS OR multimedia message service OR multi-media message OR SMS advice OR SMS reminder OR text message OR text messaging OR texting) OR AB (SMS OR short message service OR short messaging OR mobile phone messaging OR MMS OR multimedia message service OR multi-media message OR SMS advice OR SMS reminder OR text message OR text messaging OR texting) | 12,278 |
|  | TI (mobile call OR mobile calling OR mobile communication OR voice call OR voice calling OR voice message OR video conference) OR AB (mobile call OR mobile calling OR mobile communication OR voice call OR voice calling OR voice message OR video conference) | 1,751 |
|  | TI (mobile applications OR mobile apps OR mobile app OR smartphone app OR app OR apps OR email OR e-mail OR personal digital assistant OR PDA) OR AB (mobile applications OR mobile apps OR mobile app OR smartphone app OR app OR apps OR email OR e-mail OR personal digital assistant OR PDA) | 44,994 |
|  | S2 OR S3 OR S4 OR S5 | 1,759,059 |
|  | TI (eHealth OR e-Health OR electronic health OR digital health OR telehealth OR telemedicine OR telecommunication OR mHealth OR m-Health OR mobile health OR mobile medicine OR mcare OR m-care OR mobile care OR mHealth messaging OR mobile telehealth OR mobile telehealth care OR m-Edu OR medu OR m-education OR mobile education OR mLearning OR eLearning) OR AB (eHealth OR e-Health OR electronic health OR digital health OR telehealth OR telemedicine OR telecommunication OR mHealth OR m-Health OR mobile health OR mobile medicine OR mcare OR m-care OR mobile care OR mHealth messaging OR mobile telehealth OR mobile telehealth care OR m-Edu OR medu OR m-education OR mobile education OR mLearning OR eLearning) | 37,520 |
|  | S6 AND S7 | 18,351 |
|  | S1 AND S8 | 308 |

**Search history of POPLINE:**

| **SL** | **Query** | **# Hits** |
| --- | --- | --- |
|  | (expectant mother) OR (pregnant women) OR (pregnant mother) OR (pregnancy) | 65,519 |
|  | (computer) OR (tablet) OR (phone) OR (mobile) OR (mobile phone) OR (mobile device) OR (smartphone) OR (smart-phone) OR (cell phone) OR (cellphone) OR (cellular phone) OR (web) OR (website) OR (Internet) OR (online) OR (on-line) OR (technology) OR (digital technology) OR (mobile technology) OR (health technology) OR (wireless technology) OR (wireless device) OR (iPhone) OR (i-Phone) OR (iPad) OR (i-Pad) OR (iPod) OR (i-Pod) | 380,420 |
|  | (SMS) OR (short message service) OR (short messaging) OR (mobile phone messaging) OR (MMS) OR (multimedia message service) OR (multi-media message) OR (SMS advice) OR (SMS reminder) OR (text message) OR (text messaging) OR (texting) | 638 |
|  | (mobile call) OR (mobile calling) OR (mobile communication) OR (voice call) OR (voice calling) OR (voice message) OR (video conference) | 1,261 |
|  | (mobile applications) OR (mobile apps) OR (mobile app) OR (smartphone app) OR (app) OR (apps) OR (email) OR (e-mail) OR (personal digital assistant) OR (PDA) | 652 |
|  | #2 OR #3 OR #4 OR #5 | 380,420 |
|  | (eHealth) OR (e-Health) OR (electronic health) OR (digital health) OR (telehealth) OR (telemedicine) OR (telecommunication) OR (mHealth) OR (m-Health) OR (mobile health) OR (mobile medicine) OR (mcare) OR (m-care) OR (mobile care) OR (mHealth messaging) OR (mobile telehealth) OR (mobile telehealth care) OR (m-Edu) OR (medu) OR (m-education) OR (mobile education) OR (mLearning) OR (eLearning) | 4,245 |
|  | #6 AND #7 | 4,245 |
|  | (clinical trial) OR (randomized controlled trial) OR (controlled clinical trial) OR (randomization) OR (single blind procedure) OR (double blind procedure) OR (crossover procedure) OR (placebo) OR (prospective study) OR (randomised controlled trial) OR (rct) OR (randomly allocated) OR (allocated randomly) OR (random allocation) OR (single blind) OR (double blind) OR (randomized) | 17,345 |
|  | #1 AND #8 AND #9 | 133 |

**Search history of PubMed:**

| **SL** | **Query** | **# Hits** |
| --- | --- | --- |
|  | expectant mother[Text Word] OR "pregnant women"[MeSH Terms] OR pregnant women[Text Word] OR pregnant mother[Text Word] OR "pregnancy"[MeSH Terms] OR pregnancy[Text Word] | 944,178 |
|  | computer[Text Word] OR tablet[Text Word] OR phone[Text Word] OR mobile[Text Word] OR mobile phone[Text Word] OR mobile device[Text Word] OR smartphone[MeSH Terms] OR smartphone[Text Word] OR smart-phone[Text Word] OR "cell phone"[MeSH Terms] OR cell phone[Text Word] OR cellphone[Text Word] OR cellular phone[Text Word] OR web[Text Word] OR website[Text Word] OR "Internet"[MeSH Terms] OR Internet[Text Word] OR online[Text Word] OR on-line[Text Word] OR "technology"[MeSH Terms] OR technology[Text Word] OR digital technology[Text Word] OR mobile technology[Text Word] OR health technology[Text Word] OR "wireless technology"[MeSH Terms] OR wireless technology[Text Word] OR wireless device[Text Word] OR iPhone[Text Word] OR i-Phone[Text Word] OR iPad[Text Word] OR i-Pad[Text Word] OR iPod[Text Word] OR i-Pod[Text Word] | 2,423,131 |
|  | SMS[Text Word] OR short message service[Text Word] OR short messaging[Text Word] OR mobile phone messaging[Text Word] OR MMS[Text Word] OR multimedia message service[Text Word] OR multi-media message[Text Word] OR SMS advice[Text Word] OR SMS reminder[Text Word] OR text message[Text Word] OR "text messaging"[MeSH Terms] OR text messaging[Text Word]OR texting[Text Word] | 15,134 |
|  | mobile call[Text Word] OR mobile calling[Text Word] OR mobile communication[Text Word] OR voice call[Text Word] OR voice calling[Text Word] OR voice message[Text Word] OR video conference[Text Word] | 7,996 |
|  | "mobile applications"[MeSH Terms] OR mobile applications[Text Word] OR mobile apps[Text Word] OR mobile app[Text Word] OR smartphone app[Text Word] OR app[Text Word] OR apps[Text Word] OR email[Text Word] OR "electronic mail"[MeSH Terms] OR electronic mail[Text Word] OR e-mail[Text Word] OR personal digital assistant[Text Word] OR PDA[Text Word] | 179,218 |
|  | #2 OR #3 OR #4 OR #5 | 2,564,143 |
|  | eHealth[Text Word] OR e-Health[Text Word] OR electronic health[Text Word] OR digital health[Text Word] OR telehealth[Text Word] OR "telemedicine"[MeSH Terms] OR telemedicine[Text Word] OR "telecommunications"[MeSH Terms] OR telecommunication[Text Word] OR mHealth[Text Word] OR m-Health[Text Word] OR mobile health[Text Word] OR mobile medicine[Text Word] OR mcare[Text Word] OR m-care[Text Word] OR mobile care[Text Word] OR mHealth messaging[Text Word] OR mobile telehealth[Text Word] OR mobile telehealth care[Text Word] OR m-Edu[Text Word] OR medu[Text Word] OR m-education[Text Word] OR mobile education[Text Word] OR mLearning[Text Word] OR eLearning[Text Word] | 509,117 |
|  | #6 AND #7 | 184,594 |
|  | (randomized controlled trial[Publication Type] OR controlled clinical trial[Publication Type] OR randomized[Title/Abstract] OR placebo[Title/Abstract] OR clinical trials as topic[MeSH Major Topic] OR randomly[Title/Abstract] OR trial[Title]) NOT (animals[MeSH Terms] NOT humans[MeSH Terms]) | 1,114,325 |
|  | #1 AND #8 AND #9 | 659 |

**Search history of The Cochrane Library:**

| **SL** | **Query** | **# Hits** |
| --- | --- | --- |
|  | (expectant mother OR pregnant women OR pregnant mother OR pregnancy):ti,ab,kw | 39,171 |
|  | (computer OR tablet OR phone OR mobile OR mobile phone OR mobile device OR smartphone OR smart-phone OR cell phone OR cellphone OR cellular phone OR web OR website OR Internet OR online OR on-line OR technology OR digital technology OR mobile technology OR health technology OR wireless technology OR wireless device OR iPhone OR i-Phone OR iPad OR i-Pad OR iPod OR i-Pod):ti,ab,kw | 77,541 |
|  | (SMS OR short message service OR short messaging OR mobile phone messaging OR MMS OR multimedia message service OR multi-media message OR SMS advice OR SMS reminder OR text message OR text messaging OR texting):ti,ab,kw | 3,045 |
|  | (mobile call OR mobile calling OR mobile communication OR voice call OR voice calling OR voice message OR video conference):ti,ab,kw | 947 |
|  | (mobile applications OR mobile apps OR mobile app OR smartphone app OR app OR apps OR email OR electronic mail OR e-mail OR personal digital assistant OR PDA):ti,ab,kw | 4,961 |
|  | #2 OR #3 OR #4 OR #5 | 80,948 |
|  | (eHealth OR e-Health OR electronic health OR digital health OR telehealth OR telemedicine OR telecommunication OR mHealth OR m-Health OR mobile health OR mobile medicine OR mcare OR m-care OR mobile care OR mHealth messaging OR mobile telehealth OR mobile telehealth care OR m-Edu OR medu OR m-education OR mobile education OR mLearning OR eLearning):ti,ab,kw | 12,851 |
|  | #6 AND #7 | 7,380 |
|  | #1 AND #8 | 424 |

**Search history of Web of Science:**

| **SL** | **Query** | **# Hits** |
| --- | --- | --- |
|  | TI=(expectant mother OR pregnant women OR pregnant mother OR pregnancy) OR TS=(expectant mother OR pregnant women OR pregnant mother OR pregnancy) | 327,161 |
|  | TI=(computer OR tablet OR phone OR mobile OR mobile phone OR mobile device OR smartphone OR smart-phone OR cell phone OR cellphone OR cellular phone OR web OR website OR Internet OR online OR on-line OR technology OR digital technology OR mobile technology OR health technology OR wireless technology OR wireless device OR iPhone OR i-Phone OR iPad OR i-Pad OR iPod OR i-Pod) OR TS=(computer OR tablet OR phone OR mobile OR mobile phone OR mobile device OR smartphone OR smart-phone OR cell phone OR cellphone OR cellular phone OR web OR website OR Internet OR online OR on-line OR technology OR digital technology OR mobile technology OR health technology OR wireless technology OR wireless device OR iPhone OR i-Phone OR iPad OR i-Pad OR iPod OR i-Pod) | 2,023,484 |
|  | TI=(SMS OR short message service OR short messaging OR mobile phone messaging OR MMS OR multimedia message service OR multi-media message OR SMS advice OR SMS reminder OR text message OR text messaging OR texting) OR TS=(SMS OR short message service OR short messaging OR mobile phone messaging OR MMS OR multimedia message service OR multi-media message OR SMS advice OR SMS reminder OR text message OR text messaging OR texting) | 24562 |
|  | TI=(mobile call OR mobile calling OR mobile communication OR voice call OR voice calling OR voice message OR video conference) OR TS=(mobile call OR mobile calling OR mobile communication OR voice call OR voice calling OR voice message OR video conference) | 47,673 |
|  | TI=(mobile applications OR mobile apps OR mobile app OR smartphone app OR app OR apps OR email OR e-mail OR personal digital assistant OR PDA) OR TS=(mobile applications OR mobile apps OR mobile app OR smartphone app OR app OR apps OR email OR e-mail OR personal digital assistant OR PDA) | 104,785 |
|  | #2 OR #3 OR #4 OR #5 | 2,089,297 |
|  | TI=(eHealth OR e-Health OR electronic health OR digital health OR telehealth OR telemedicine OR telecommunication OR mHealth OR m-Health OR mobile health OR mobile medicine OR mcare OR m-care OR mobile care OR mHealth messaging OR mobile telehealth OR mobile telehealth care OR m-Edu OR medu OR m-education OR mobile education OR mLearning OR eLearning) OR TS=(eHealth OR e-Health OR electronic health OR digital health OR telehealth OR telemedicine OR telecommunication OR mHealth OR m-Health OR mobile health OR mobile medicine OR mcare OR m-care OR mobile care OR mHealth messaging OR mobile telehealth OR mobile telehealth care OR m-Edu OR medu OR m-education OR mobile education OR mLearning OR eLearning) | 97,633 |
|  | #6 AND #7 | 53,396 |
|  | TI=(clinical trial OR randomized controlled trial OR controlled clinical trial OR randomization OR single blind procedure OR double blind procedure OR crossover procedure OR placebo OR prospective study OR randomised controlled trial OR rct OR randomly allocated OR allocated randomly OR random allocation OR single blind OR double blind OR randomized) OR TS=(clinical trial OR randomized controlled trial OR controlled clinical trial OR randomization OR single blind procedure OR double blind procedure OR crossover procedure OR placebo OR prospective study OR randomised controlled trial OR rct OR randomly allocated OR allocated randomly OR random allocation OR single blind OR double blind OR randomized) | 1,294,281 |
|  | #1 AND #8 AND #9 | 280 |
